# Removal of Vanadium(III) and Molybdenum(V) from Wastewater Using *Posidonia oceanica* (Tracheophyta) Biomass

**DOI:** 10.1371/journal.pone.0076870

**Published:** 2013-10-25

**Authors:** Chiara Pennesi, Cecilia Totti, Francesca Beolchini

**Affiliations:** Department of Life and Environmental Sciences, Polytechnic University of Marche, Ancona, Italy; Queen's University Belfast, United Kingdom

## Abstract

The use of dried and re-hydrated biomass of the seagrass *Posidonia oceanica* was investigated as an alternative and –low-cost biomaterial for removal of vanadium(III) and molybdenum(V) from wastewaters. Initial characterisation of this biomaterial identified carboxylic groups on the cuticle as potentially responsible for cation sorption, and confirmed the toxic-metal bioaccumulation. The combined effects on biosorption performance of equilibrium pH and metal concentrations were investigated in an ideal single-metal system and in more real-life multicomponent systems. There were either with one metal (vanadium or molybdenum) and sodium nitrate, as representative of high ionic strength systems, or with the two metals (vanadium and molybdenum). For the single-metal solutions, the optimum was at pH 3, where a significant proportion of vanadium was removed (*ca.* 70%) while there was ca. 40% adsorption of molybdenum. The data obtained from the more real-life multicomponent systems showed that biosorption of one metal was improved both by the presence of the other metal and by high ionic strength, suggesting a synergistic effect on biosorption rather than competition. There data ware used for the development of a simple multi-metal equilibrium model based on the non-competitive Langmuir approach, which was successfully fitted to experimental data and represents a useful support tool for the prediction of biosorption performance in such real-life systems. Overall, the results suggest that biomass of *P. oceanica* can be used as an efficient biosorbent for removal of vanadium(III) and molybdenum(V) from aqueous solutions. This process thus offers an eco-compatible solution for the reuse of the waste material of leaves that accumulate on the beach due to both human activities and to storms at sea.

## Introduction

*Posidonia oceanica* (L.) Delile, is the most important and abundant seagrass and it is endemic to the Mediterranean Sea. It forms large underwater meadows from the surface to depths of 40 m, which are an important part of the ecosystem [1], [2] *P. oceanica* has a significant ecological role, as it can form structures known as ‘*matte*’, which are monumental constructions that result from the horizontal and vertical growth of the rhizomes with their entangled roots and the entrapped sediment [3]–[5]. This seagrass is very sensitive to human disturbance, such as coastal development, pollution, trawling and high water turbidity [3]–[5]. Indeed, in the year 2000, *P. oceanica* was selected as a Biological Quality Element [6] under the Water Framework Directive [7], as a representative of the aquatic Mediterranean angiosperms for use in the monitoring of the ecological status of coastal waters. *P. oceanica* in Italy is mostly present along the Tyrrhenian and Ionian coasts, where it is destroyed mainly by trawling and by high water turbidity [8]. Human activities and sea storms result in the accumulation of the leaves of this plant on beaches; and their disposal represents a significant environmental problem [9]. This can, however, be avoided if this waste material can be transformed into a resource.

The physicochemical process known as ‘*biosorption*’ indicates the removal of heavy metals from an aqueous solution by passive binding to non-living biomass [10]–[12]. Marine biomass represents an important resource for biosorption processing of heavy metals from industrial wastewater. Indeed, non-living seaweed and seagrass can be used as low-cost sorbents as an alternative to more costly synthetic resins [10]–[16].

The high metal-binding capacity of seaweeds is due to the structure of their cell wall, with various functional groups involved. These include: (1) alginic acid, with carboxyl groups and sulfated polysaccharides, such as fucoidan, and with sulfonic acid, in brown algae matrix (Phylum Ochrophyta) [10], [11], [17]; (2) sulfated galactans such as agar, carregeenan, porphyran, and furcelleran in red algae (Phylum Rhodophyta) [10], [12], [18]; (3) an external capsule that is composed of proteins and/or polysaccharides in green algae (Phylum Chlorophyta) [19]. While algal materials have been broadly investigated for metal biosorption (Table 1), the potential of marine plants has been notably understudied. However, recent studies in the literature have stated that the ability of seagrasses to adsorb heavy metals also depends on the chemical structure of the plant tissues [11], [12], [20], [21]. In particular, Pennesi at al. [11], [12] studied for the first time the biosorption performance of *Cymodocea nodosa* (Ucria) Ascherson and *Zostera marina* Linnaeus, for the removal of lead and arsenic from aqueous solution. They showed that the optimal biosorption of heavy metal occurs because of the chemical composition of the thin cuticle which is the external layer that covers the leaves. Cutin is a waxy polymer that is the main component of the plant cuticle, and it consists of omega hydroxy acids and their derivatives, which are interlinked via ester bonds, to form a polyester polymer of indeterminate size [22]. This substance is probably responsible for the chemical and physical bond with heavy metals, through the carboxylic groups.

**Table 1:** Sorption performance by marine macrophytes according to the literature.

Macrophyte	Metal	q (mg/g)	C (mg/L)	Conditions	References
*Fucus vesciculosus*	Cr(III)	63	52–78	pH 4.5 T25°C	Murphy et al. (2008)
	Cr(VI)	44	52–78	pH 2	Murphy et al. (2008)
	Cd	73	-	-	Holan et al. (1993)
*Ascophlyllum nodosum*	Cu+Pb+Zn+Ni	117	0.5–1	pH 4	Zhang and Banks (2006)
	Pb	280	200	pH 6,T25°C	Veglio and Beolchini (1997)
	Au	24	-	pH 2.5	Thomas et al. (2003)
	Cd	214	-	pH 4.9	Thomas et al. (2003)
	Co	100	-	pH 4.0	Thomas et al. (2003)
*Fucus spiralis*	Cr(III)	61±5	26–52	pH 4.5	Murphy et al. (2008)
	Cr(VI)	35.3±3	52–78	pH 2	Murphy et al. (2008)
	Cd	146	-	-	Cordeo et al. (2004)
*Ulva lactuca*	Cr(III)	26.2±2	52–78	pH 4.5	Murphy et al. (2008)
	Cr(VI)	28±4	78–104	pH 2	Murphy et al. (2008)
*Ulva spp.*	Cr(III)	53±5.2	52–78	pH 4.5	Murphy et al. (2008)
	Cr(VI)	30±4	52–78	pH 2	Murphy et al. (2008)
*Palmaria palmata*	Cr(III)	30±2	52–78	pH 4.5	Murphy et al. (2008)
	Cr(VI)	34±5	52–78	pH 2	Murphy et al. (2008)
*Polysiphonia lanosa*	Cr(III)	34±2	52–78	pH 4.5	Murphy et al. (2008)
	Cr(VI)	46±6	52–78	pH 2	Murphy et al. (2008)
*Laminaria japonica*	Pb(II)	286	300–400	pH 4.1	Ghimire et al. (2008)
	Cd(II)	128	200–400	pH 4.1	Ghimire et al. (2008)
	Fe(III)	49.28	100–200	pH 4.1	Ghimire et al. (2008)
	Ce(III)	123	200–400	pH 4.1	Ghimire et al. (2008)
	Zn	91	-	pH 4.5	Thomas et al. (2003)
*Sargassum fluitans*	Cu	112	-	-	Kratochvil et al. (1997)
	Cd	79.52	-	pH 4.5	Thomas et al. (2003)
*Sargassum natans*	Cd	135	-	pH 3.5 T 26°C	Holan et al. (1993)
	Au	400	79–120	pH 2.5	Thomas et al.(2003)
*Sargassum hemiphyllum*	Cr(III)	72.2	-	-	Murphy et al. (2008)
*Spirogyra*	Cr(III)	28	-	-	Murphy et al. (2008)
	Pb	1449	-	pH 3.5 T 26°C	Murphy et al. (2008)
*Halimeda opuntia*	Cr(III)	40	-	pH 4.1 T 26°C	Veglio and Beolchini (1997)
*Cystoseira compressa*	Pb	11	-	room temperature	Pennesi et al. (2012a)
*Scytosiphon lomentaria*	Pb	68	-	room temperature	Pennesi et al. (2012a)
*Ulva rigida*	Pb	30	-	room temperature	Pennesi et al. (2012a)
*Ulva compressa*	Pb	46	-	room temperature	Pennesi et al. (2012a)
*Gracilaria bursa-pastoris*	Pb	19	-	room temperature	Pennesi et al. (2012a)
*Porphyra leucosticta*	Pb	68	-	room temperature	Pennesi et al. (2012a)
*Polysiphonia* sp.	Pb	68	-	room temperature	Pennesi et al. (2012a)

*P. oceanica* not only contains cutin, but it is also a highly fibrous material that is made of cellulose and hemicellulose (*ca.* 60%–75%) and lignin (*ca.* 25%–30%), plus a relevant percentage of ash that contains essentially silica and traces of some heavy metals [23]. Moreover, *P. oceanica* contains two type of metallothioneins (MTs) which are a group of cysteine-rich proteins. These proteins have the capacity to bind heavy metals through the thiol groups, which are also known as sulfhydryl groups (R-SH), and amino groups (R-NH_2_) of its cysteine residues [24], [25]. Other studies have investigated the biosorption of heavy metals such as copper, lead, and chromium, and the removal of dyes from textile waters using *P. oceanica* biomass [21], [26]–[30].

Vanadium and molybdenum are discharged into the environment from various industries that work on alloy steels [31], [32], and these are considered to be persistent environmental contaminants [33], [34]. Due to their toxicity and their accumulation throughout the food chain, they represent a significant problem with ecological and human-health effects. Thus, it is appropriate to eliminate these heavy metals from industrial wastewaters using cheap material such as marine macrophytes [10]–[12], [18], [19], [35].

In the present study, the *P. oceanica* biomass, consisted of dried leaves (material from a beach) that was used for the first time as a low-cost biosorbent for the removal of vanadium(III) and molybdenum(V) from aqueous solution. The objectives of this study were (1) to evaluate the performance of *P. oceanica* non-living biomass in ideal single-metal systems (with either vanadium or molybdenum) under different pH conditions (to determine the optimum operating conditions); (2) to determine any competition phenomena in more real-life systems that were characterized by either high ionic strength or the presence of both vanadium and molybdenum; and (3) to define an equilibrium model to predict the biosorption performance under these more real-life conditions.

## Materials and Methods

### Sampling and preparation

The biosorbent materials was obtained from dead leaves of *P. oceanica* (Phylum: Tracheophyta). The samples were collected from Italian beaches of the Ionian Sea: Marina di Leporano, Taranto (Puglia Region; 40°21′43.60″N, 17°20'00.05″E). Specific permission was not required for this geographical location because it is not part of the Marine Protected Area. Furthermore, the material used was destined for waste disposal. After collection, the samples were washed in deionized water (10% w/v, aquaMAXTM, Basic 360 Series) to remove the remaining salt and impurities. Subsequently, the biomass was washed in acid solution (HCl 0.1 N, pH 2; Carlo Erba Reagents) for 4 h under vigorous stirring at room temperature (ratio solid/washing solution 1/10) to remove any traces of metals from the binding sites of the seagrass. Then the leaves were dried (Dry Systems Labconco, Kansas City, MO) at room temperature for 4–5 days to 5 days (to a stable weight) and kept in bottles (Kartell) until use. Before the biosorption tests, each dried biomass sample was reduced into small fragments (of about 0.5 cm, Porcelain Mortar & Pestle Carlo Erba, model 55/8) and re-hydrated before use to increase the percentage removal. This field study did not involve any endangered or protected species.

### Reagents

Stock solutions of molybdenum(V) chloride (MoCl_5_, Sigma-Aldrich®) and vanadium(III) chloride (VCl_3_, Sigma-Aldrich®) at, 1 g/L were prepared in distilled water (Sigma-Aldrich®). All of the working solutions at the various concentrations were obtained by successive dilution, according the experimental design (see section 2.5).

### Characterization of functional groups: titration test

The characterization of the functional groups on *P. oceanica* that are involved in binding the metals was carried out by acid-base titration test and analyzed according to the Gran Method [36], [37]. Biosorbent materials (i.e, 5 g leaves of *P. oceanica* in 100 mL deionised water) were titrated using standard solutions of NaOH 1 N (basic branch, Sigma-Aldrich®) and H_2_SO_4_ 0.1 N (acid branch, Sigma-Aldrich®). The pH of the suspension was measured after each addition of titrant (0.05 mL, Eppendorf Research® plus) when stability had been obtained, using a pH-meter (ISteK pH 730p).

### Adsorption tests

Before each test, 5 g dried *P. oceanica* biomass was put into 100 mL distilled water for 30 min to rehydrate the sample, with stirring using a magnetic stirrer (MICROSTIRRER-VELP Scientific). The concentrated metal stock solutions (1 g/L molybdenum; 1 g/L vanadium) were added according to the experimental conditions. The suspension pH was adjusted with HCl (0.1 M) and NaOH (0.1 M) and monitored during the whole biosorption test. Aliquot amounts of the solution (1 mL) were periodically sampled to determination the metal concentration(s). The samples were centrifuged (3000 rpm for 5 min.; ALC centrifuge PK 120), to eliminate any suspended matter before the analytical determination, and they were subsequently diluted with acidified water at pH 2 to stabilize the metal(s) before the analytical determinations. Control tests were performed without biomass and showed constant metal concentrations in the solutions over time. This confirmed that precipitation did not take place and that no metal was released by the testing equipment. Metal uptake, *q* (mg g^−1^), was calculated as the difference in the metal concentration(s) in the aqueous phase before and after sorption, according to Eq. A:
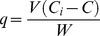
(A)where, *V* is the volume of the solution (L), *C_i_* and *C* are the initial and equilibrium concentration of metal (molybdenum or vanadium) in solution (mg L^−1^), respectively, and *W* is the mass of the dry leaves (g). Through regression analysis, the Langmuir adsorption isotherm [38] given in Eq. (B) was adapted to the experimental data:
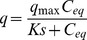
(B)where *q_max_* is the *maximum adsorption* (mg/g) and Ks is the equilibrium constant of the sorption reaction (mg/L).

### Experimental design

Table 2 shows the factors and levels investigated for the biosorption of these leaves of *P. oceanica*. All of the experiments were carried out at a constant room temperature. For the ideal systems and the real-life systems with high ionic strength, the equilibrium pH was the only factor considered in the experimental plan. In particular, for the ideal single metal systems, sorption isotherms were evaluated at pH 1, 2, 3, 6, 8, 10 and 12 for vanadium, and at pH 1–3, 5, 7, 9, 10 and 12 for molybdenum. For high ionic strength systems, 20 mg/L NaNO_3_ was added at the beginning of each experiment, and the sorption isotherms were evaluated at pH 3, 6 and 9 for vanadium, and at pH 6, 9 and 12 for molybdenum,. In the case of the more real-life multi-metal systems, the sorption isotherms were determined at pH 3, and the only factor considered in the experimental plan was the presence of the other metal (0,20 and 40 mg/L vanadium/molybdenum for the molybdenum/vanadium sorption, respectively).

**Table 2:** Factors and levels investigated in the study of molybdenum(V) and vanadium(III) biosorption by biomass of *Posidonia oceanica*.

Experimental system	Factorial plan		
	Factors	Levels	
Ideal single metal system	Metals	Mo(V)	V(III)
	pH	1–3; 6; 8; 10; 12	1–3; 5; 7; 9; 10; 12
Real life high ionic strength (single metal system with 20 mg/L NaNO_3_)	Metals	Mo(V)	V(III)
	pH	6; 9; 12	3; 6; 9
Real life two-metal system	Metal 1	Mo(V)	V(III)
	Metal 2	absent; 20 mg/L; 40 mg/L	absent; 20 mg/L; 40 mg/L

### Analytical determinations

The pH measurements were made using a pH meter (ISteK 730p). All of the samples were diluted with HNO_3_ at pH 2 and stored at 4°C before analysis. The metals concentrations in the liquid phase were determined by ICP-AES (Inductively Coupled Plasma Atomic Emission Spectrometry) (Jobin Yvon JY 24, method EPA200.7.2001).

## Results and Discussion

### Biomass characterization

#### *Posidonia oceanica* characterisation: acid-base titration

**Figure 1:**
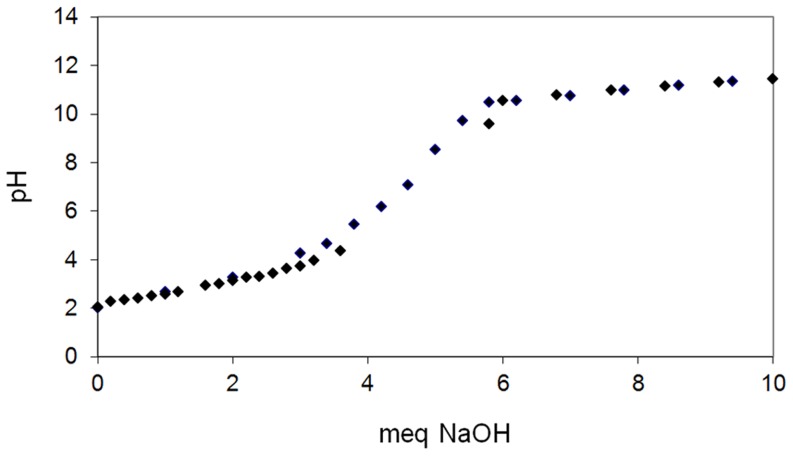
*P. oceanica* titration curve (biosorbent 10 g/L; room temperature).

The number and type of functional groups involved in the binding of the metals onto the *P. oceanica* samples were analyzed using acid-base titration [36], [37], [39]. This analysis is based on a neutralization reaction, in order to determine an unknown concentration of functional groups that have an acid behaviour in aqueous solution. The *P. oceanica* titration curve in Figure 1 shows the pH profile as a function of the added m_eq_ NaOH: (1) this curve is typical of weak polyprotic acids, which have more than one proton that can be removed by reaction with a base; (2) the equivalence point (i.e., where all of the protons are neutralized by the added hydroxylic groups) is not easily identifiable. The Gran method was used to linearise the titration curve before and after the equivalence point (Fig. S1). The volume necessary to reach the equivalence point was estimated as in the range of 3500–5000 µL (Fig. S1), which when considering the biomass of *P. oceanica* in solution (i.e., 10 g/L), corresponds to a concentration of functional groups with an acid behavior of 3.5–5 m_eq_/g biomass. It can be seen that this titration procedure allowed the estimation of a range for the volume of equivalence, rather than a single value, as in the case of titration of pure acid solutions. This can be explained by considering the heterogeneity of the solid matrix subjected to titration, in terms of these dead leaves of *P. oceanica*. Furthermore, the titration curve in Figure 1 allows an estimation of the acid dissociation constant (*K_a_*) of these functional groups that behave as weak acids. Indeed, the *pKa* (i.e., the pH corresponding to half the equivalence point) [40], is in the range of 3 to 4, which is typical of the R–COO− carboxyl groups of the *P. oceanica* cuticle [17]. Similar results were also obtained for the carboxyl groups in the biomass of the algae *Chlorella pyrenoidosa* H. Chick, *Cyanidium caldarium* (Tilden) Geitler [41] and *Sargassum fluitans* (Børgesen) Børgesen [42]. Moreover, previous studies have used Fourier transform infrared spectroscopy analysis (FTIR) to demonstrate that the functional groups of *P. oceanica* that might have a role in the adsorption process are carboxyl (COOH) and carbonyl stretching (C = O) groups in particular [28], [29].

#### Heavy metal concentration in *Posidonia oceanica* leaves

The leaves of *P. oceanica* show significant concentrations of As, Hg, Mo, Pb and V in their tissues (Table 3). Before metals determination, a part of the stock was first washed in acid at pH 2 to remove metals eventually bound at the surface level, while another sample was analyzed under natural conditions. It can be seen that there were no significant differences between the metal concentrations in the two samples (Table 3). This suggests that the *P. oceanica* tissue contains traces of metals, which will be connected to the phenomenon of bioaccumulation, of which there are many studies in the literature [43]–[47]. This result might be related to the presence of industrial sites (e.g., refinery, metallurgical plant and commercial port) located near the collection site (about 30 km away). Indeed, *P. oceanica* is increasingly used as an indicator of chemical contaminations for coastal regions of the Mediterranean Sea, and it is often considered a useful metal bio-indicator [44], [45], [48]. In particular, *P. oceanica* has been investigated as a bio-indicator for mercury, which was also found in samples used for this study (Table 3), and some studies have suggested that several trace metals can be memorized through analysis of its below-ground tissues [44], [49], [50].

**Table 3:** Heavy metal concentrations in the samples of *Posidonia oceanica* leaves.

Metal	*P. oceanica* (mg Kg^−1^) acid wash (pH 2)	*P. oceanica* (mg Kg^−1^) no acid wash
arsenic	2	5
mercury	0.12	0.15
molybdenum	2.5	1.6
lead	2.4	3
vanadium	12	14

### Vanadium and molybdenum speciation

**Figure 2:**
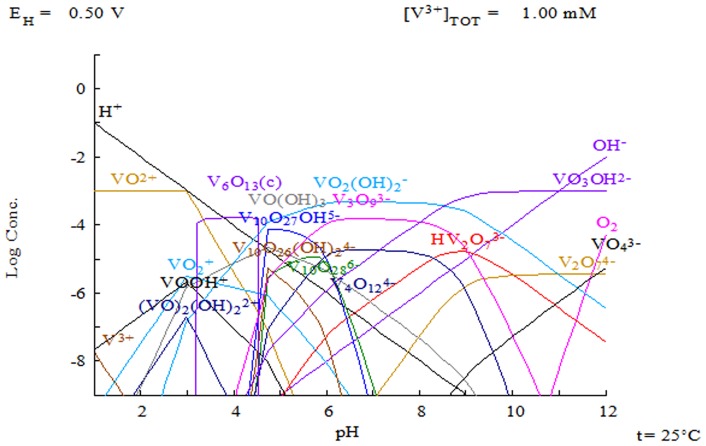
Prediction of vanadium(III) speciation as a function of pH where y-axis shows the logarithm of the metal concentration (MEDUSA software) [51].

**Figure 3:**
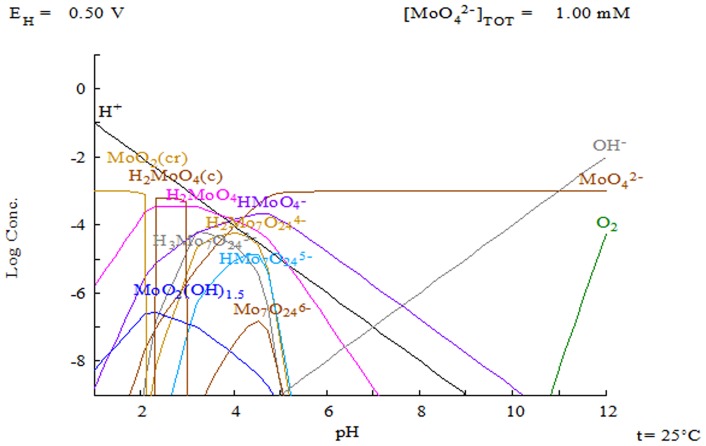
Prediction of molybdenum(V) speciation as a function of pH where y-axis shows the logarithm of the metal concentration (MEDUSA software) [51].

In this section, vanadium(III) and molybdenum(V) speciation as a function of pH is discussed, in terms of the predictions of the MEDUSA software [51]. The chemistry of vanadium and molybdenum is complex; indeed, such metals can be present in solution in both anionic and cationic forms. For this reason, theoretical predictions can help both in the choice of experimental conditions to be investigated and in the discussion of the results obtained. Figures 2 and 3 show the predictions for vanadium(III) and molybdenum(V) speciation as a function of pH as given by the MEDUSA software, where the x-axis indicates the pH while the y-axis shows the logarithm of the metal concentration. It can be seen that anionic forms, cationic forms and insoluble species can be present under different pH conditions (Figs 2, 3). The pH range where vanadium appears to be stable in its ionic form in solution is wide (Fig. 2); indeed, vanadium speciation includes both cationic forms (for pH 2–6) and anionic forms (from pH 5 onwards). Consequently, both negatively (e.g., carboxylic groups) and positively (e.g., aminic groups) charged sites of *P. oceanica* biomass are considered to be involved in vanadium biosorption. For molybdenum, Figure 3 shows that it is mainly stable in solution in an anionic form for pH>3; this suggests that positively charged binding sites on the *P. oceanica* are responsible for molybdenum biosorption.

### Biosorption: the ideal single metal system

**Figure 4:**
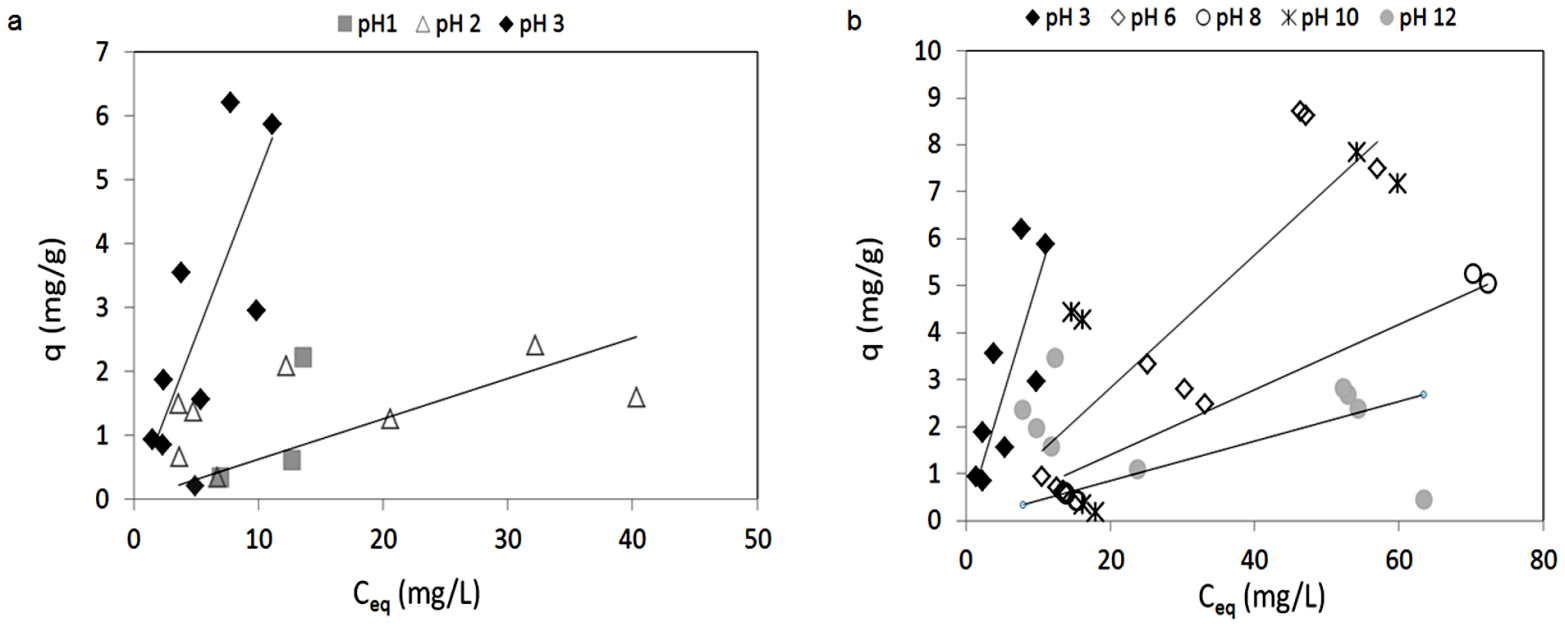
Sorption isotherms for vanadium in the single metal system, for the range of pH–3 (a) and 3–12 (b) (biosorbent 10 g/L; room temperature).

**Figure 5:**
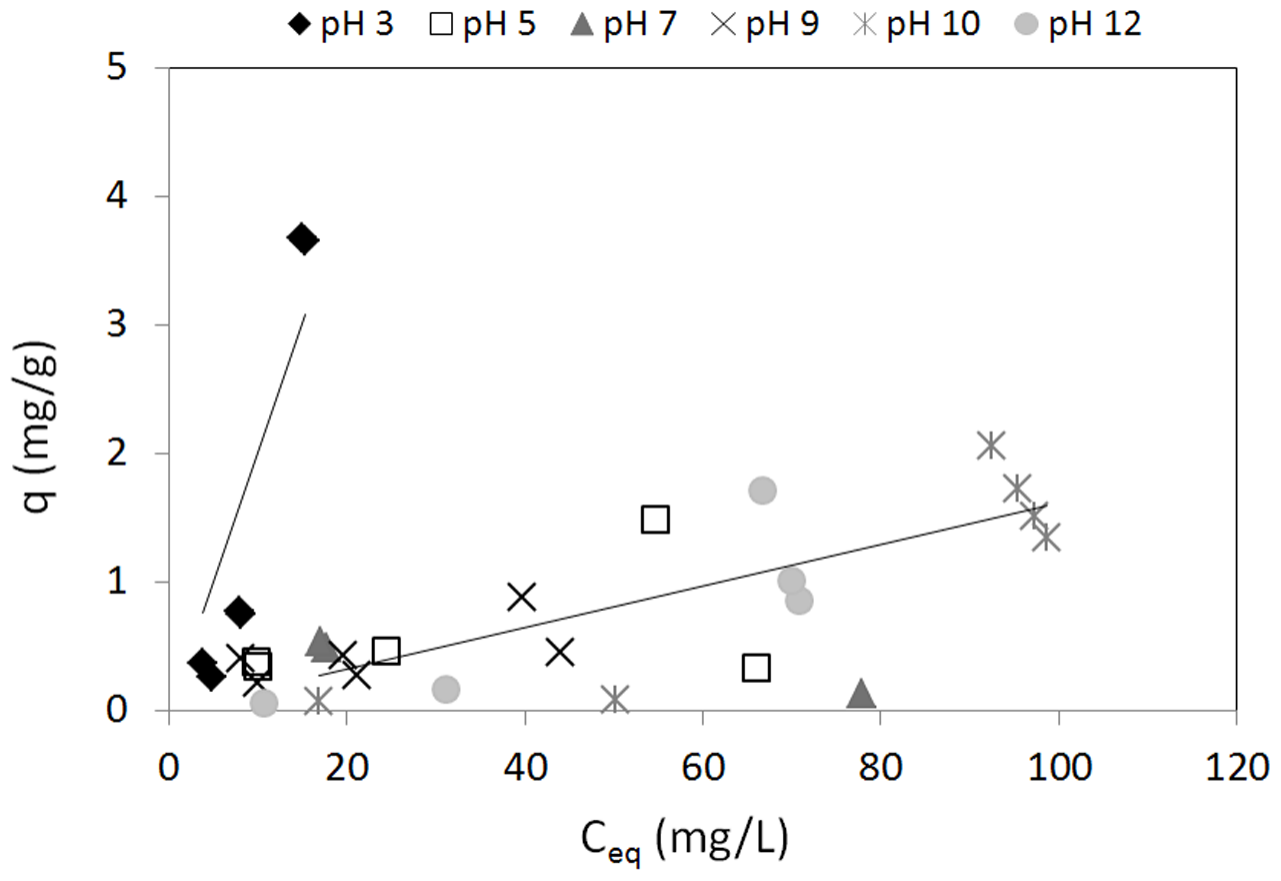
. Sorption isotherms for molybdenum in the single metal system, for the range of pH 3–12 (biosorbent 10 g/L; room temperature).

This part of the study was aimed at an evaluation of the influence of pH on vanadium and molybdenum biosorption by *P. oceanica* in the single metal systems (Table 2; two levels investigated). Figure 4 shows the sorption isotherms for vanadium that were determined for the different equilibrium pHs (pH 1–3, Fig. 4a; pH 3–12, Fig. 4b). It can be seen that the optimal pH for vanadium biosorption by *P. oceanica* is pH 3, with *maximum adsorption* of about 7 mg/g with an equilibrium concentration of vanadium in solution of 10 mg/L (Fig. 4a). The profiles in Figure 5 also show that the adsorption capacity decreases at both pH<3(Fig. 4a) and >3 (Fig. 4b). Furthermore, control experiments with no biomass excluded any significant precipitation phenomena. For molybdenum biosorption in the single metal ideal systems (Fig. 5), the best adsorption performance was at pH 3 with *maximum adsorption* around 4 mg/g with the molybdenum equilibrium concentration of 10 mg/L (Fig. 5). Significant precipitation occurred at pH 1 and 2, as also predicted theoretically by the MEDUSA software [51] (Fig. 3). For the pH range from pH 5 to 12, the highest observed values for the specific uptake (q) were around 2 mg/g.

### Biosorption: real life multicomponent systems

#### High ionic strength systems

**Figure 6:**
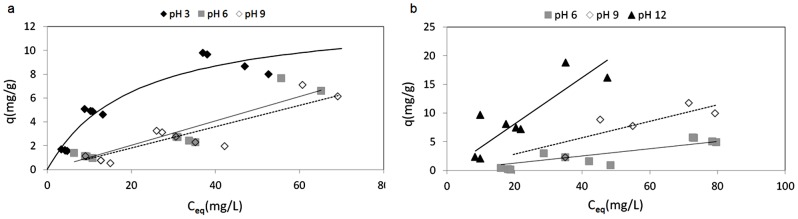
Sorption isotherms in real life high ionic strength systems: pH effect for vanadium (a) and molybdenum (b) in the presence of NaNO_3_ 20 mg/L (biosorbent 10 g/L; room temperature).

This part of the study was dedicated to the estimation of the influence of pH on vanadium and molybdenum biosorption by *P. oceanica* in the presence of NaNO_3_ (20 mg/L). Sodium nitrate was added (Table 2) to simulate real-life systems, by providing potential antagonist ions that can compete with the metals for the active sites involved in biosorption on the cuticle of *Posidonia oceanica*. The solution was diluted according to the experimental design (see section 2.5; Table 2; two levels investigated). Figure 6 shows the effects of pH on the sorption isotherms for vanadium and molybdenum, respectively, in the presence of the antagonist ions (NaNO_3_). A comparison with the data observed in the single metal systems (Fig. 4), confirms that pH 3 was optimal for the adsorption of vanadium (Fig. 6a) and that no competition appears to have taken place. The data in Figure 6b suggest that the adsorption capacity for molybdenum was optimal at pH 12, while in the single metal system, no significant adsorption was observed at this pH (Fig. 5). The oxidative action of NaNO_3_ might have modified the speciation of molybdenum (Fig. 3), and then modified the performances at pH 3. These data show that there are linear correlations between the metal concentrations on the solid and in the liquid in equilibrium, except for the adsorption of vanadium at pH 3, which appears to follow a Langmuir trend.

#### Multi-metal systems

**Figure 7:**
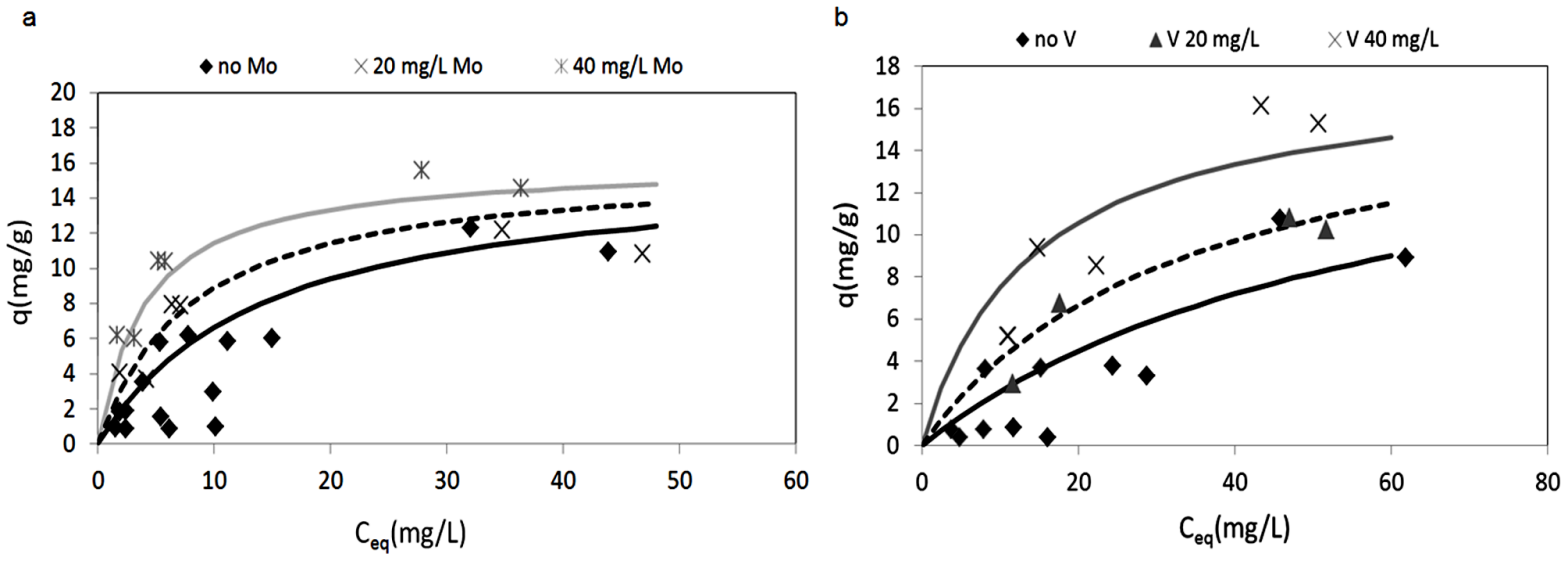
Sorption isotherms in the two metal system for vanadium (a) and molybdenum (b) in the presence of the other metal respectively (pH 3; biosorbent 10 g/L; room temperature).

Potential competition between vanadium and molybdenum might affect the biosorption performances when these metals are present simultaneously. The sorption isotherms were determined at pH 3 considering that this pH was the optimum for the biosorption performances of both of these metals, in the ideal single metal systems. Figure 7 shows the sorption isotherms of each metal in the presence of increasing concentration of the other metal in solutions diluted according to the experimental design (see section 2.5; Table 2; two levels investigated). It can be seen that no competition appeared to take place, and that conversely, the presence of one of the metals appears to favor the adsorption of the other metal. The absence of competition can be explained with reference to theoretical predictions previously reported (see section 3.2): at *ca.* pH 3, molybdenum is mainly stable as an anion, while vanadium is a cation. Consequently, this indicates that bothof these ions can be adsorbed simultaneously; as they have opposite charges, the two metals interact with different binding sites on the *P. oceanica* cuticle. The Langmuir equation of Eq. (B) was fitted to the experimental data, and Table 4 gives the estimated values for the parameters *q_max_* and *Ks*. Here, it can be seen that parameter *q_max_* is estimated at 16 mg/g and 18 mg/g for vanadium and molybdenum, respectively, without any significant effects of the other metal; on the other hand, parameter *Ks* significantly decreased as the concentration of the other metal increased. This trend confirmed the absence of competition between these two metals, and suggested that the identification of a mathematical model suitable for predicting real multi-metal systems should take into account the variability of the sorption equilibrium constant, *Ks*, depending on the concentration of the other metal. This approach is described in the following section.

**Table 4:** Langmuir model parameters in the multi-metals systems (vanadium and molybdenum).

	vanadium – pH 3			molybdenum – pH 3		
	no Mo	20 mg/L Mo	40 mg/L Mo	no V	20 mg/L V	40 mg/L V
**q_max_ (mg/g)**	16	16	16	18	18	18
**k_s_ (mg/L)**	14	8	4	60	34	14
**R^2^**	0.9	0.95	0.96	0.9	0.95	0.93

#### Mathematical modeling of multi-metal equilibrium

As reported above, the theoretical prediction of vanadium speciation (Fig. 2) suggests that under conditions of pH 3, vanadium is substantially present as the VO_2_^+^ cation. Consequently the biosorbent functional groups involved in vanadium sorption are considered to be carboxylic groups (as documented in the literature [28] and shown by the acid-base titration), according to the following simplified sorption/ion-exchange mechanism:
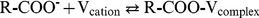
(C.1)where the carboxylic groups are dissociated in the water solution according to the following acid dissociation equilibrium:
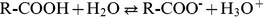
(C.2)with a pK_A_ in the range of 3 to 4 as previously reported.

For molybdenum, the theoretical prediction of its speciation (Fig. 3) indicates that at pH 3 it is mainly present as the negatively charged species: H_3_Mo_7_O_24_^3−^ and MoO_4_^2−^, where this latter becomes the ion dominant species as the pH increases. Contrary to what happens for vanadium, in this case, the biosorbent functional groups involved in molybdenum sorption are considered to be positively charged groups, as mainly aminic groups, which are well known as being present on the seagrass cuticle [22], according to the following mechanism:
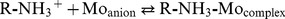
(C.3)


Where, the aminic groups are dissociated in water solution according to the following acid dissociation equilibrium:
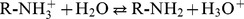
(C.4)with a pK_A_ in the range 9–10 [52].

In summary, the vanadium and molybdenum sorption process is relatively complex, with many factors involved, such as the metal speciation in solution and the dissociation equilibria of the main functional groups in the biosorbent material. Considering the *pKa* values reported above, it is expected that under pH 3, at least half of the carboxylic functional groups are available for vanadium biosorption, while all of the aminic groups are positively charged and ready to bind molybdenum complexes. To define a simple mathematical model that can be used to predict the sorption of one metal in the presence of the other, was taken into account that the two metals do not compete for the same sites, due to the opposite charges of the species involved. Consequently, in this case a non-competitive Langmuir equilibrium model can be considered as the simplest one to be tested for data fitting, as follows:
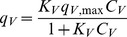
(C.5)

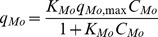
(C.6)where *C_V_* and *C_Mo_* represent the equilibrium concentrations in solution, *q_V_* and *q_Mo_* are the metal specific uptakes, *q_V,max_* and *q_Mo,max_* are the maximum metal-specific uptakes and *K_V_* and *K_Mo_* are the equilibrium constant of sorption in Eqs. (C.1) and (C.3), respectively.

The experimental data reported in Figure 7 shows that for both of these metals, the presence of the other metal favors the biosorption: e.g., vanadium sorption in the presence of molybdenum is higher than in the single metal system. This can be explained by a partial neutralization of charged sites that might have a repulsing action; consequently, in the presence of molybdenum, the positively charged aminic groups are neutralized, and their repulsion towards the positively charged vanadium ions is removed. For molybdenum biosorption an analogous consideration can be performed, as the opposite effect. To take these phenomena into account in the sorption model, the presence of one metal increases the equilibrium constant of the other metal sorption according to the following empirical rules:
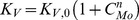
(C.7)

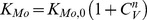
(C.8)


**Figure 8:**
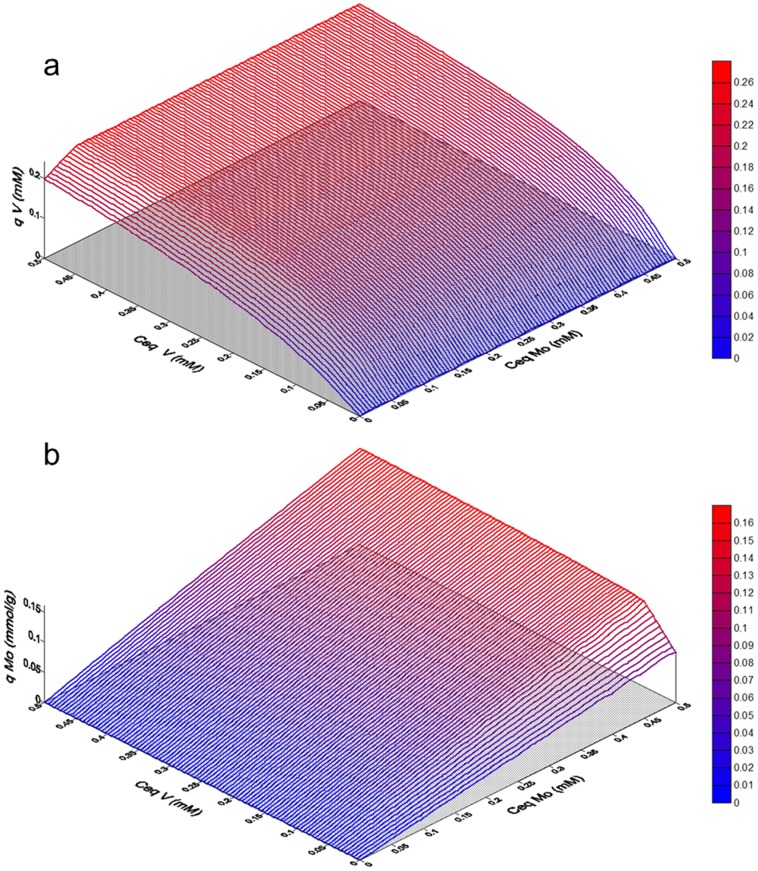
Sorption isotherms of vanadium (a) and molybdenum (b) as a function of the two metal concentrations, as predicted by equations (C.5) and (C.6) (pH 3, parameters as in Table 5).

Equations (C.5) and (C.6), with the equilibrium constants given by equations (C.7) and (C.8), were fitted to the experimental data by non linear regression analysis for parameter estimation, through the least-squares method. Table 5 shows the estimated values for the parameters and the performance of the data fitting, which can be considered satisfactory considering that the multiple regression coefficient R2 is near 0.90. Figure 8 shows the equilibrium specific uptake of vanadium (Fig. 8a) and molybdenum (Fig. 8b) as functions of the two metal concentrations, as predicted by equations (C.5) and (C.6), where the synergic effects of the two metals on the biosorption performance can be seen.

**Table 5:** Estimated parameters for the synergistic equilibrium sorption isotherms from Eqs. (C.5) and (C.6).

**q_V, max_**	0.31 mmol/g
**K_V, 0_**	3.5 L/mmol
**n**	0.02
**q_Mo, max_**	1.3 mmol/g
**K_Mo, 0_**	0.14 L/mmol
**m**	0.12
**R^2^**	0.89

## Conclusion

The disposal of biomass of *Posidonia oceanica* accumulated on the beaches represents a significant environmental problem [9], that could be avoided if such waste material is transformed as a resource. Some few examples are available in the literature where its potential use as metal biosorbent is assessed: indeed, it has already been demonstrated that *Posidonia oceanica* could adsorb uranium (VI) [53], chromium (VI) [54], lead (II) [28] with the highest sorption capacities of 5.67, 14.48 and 140 mg/g, respectively. Furthermore this biomass has also been reported to effectively adsorb anionic species, such as ortophosphate ions, with sorption capacity of 7.45 mg/g [55]. This work demonstrates the suitability of such biomass also as vanadium and molybdenum sorbent, with a maximum sorption ability estimated at 16 and 18 mg/g, respectively. Sorption of vanadium and molybdenum was explained by chemico-physical interaction (mainly based on ion exchange) with carboxilic and amminic groups that are present in many macromolecules on the cuticle of the plant (e.g. cutin, metallothionein [22], [24], [25]). The real system simulation allowed to exclude any competition phenomena of nitrate ions and of one metal with the other, due to a different speciation of vanadium and molybdenum in aqueous solution (cation vs. anion). This evidence has allowed to develop a new simple multi-metal sorption equilibrium model that is able to take into account the synergic effect on the biosorption performance, that was evident when both metals were present. The availability of a mathematical tool that can predict the performance of biosorption in such multi-metal systems is considered very important [56], [57] and there is a real need that new scientific literature goes beyond the very well-known Langmuir/Freundlich sorption models, representative of ideal single metal systems. Future work will be addressed on one hand at real systems coming from refinery catalysts recycling process [58], [59], on the other hand at the upscale of the biosorption process, in order to find a suitable process configuration for the application of *Posidonia oceanica* biomass at industrial scale.

## Supporting Information

Figure S1 Gran elaboration of *P. oceanica* titration curve (biosorbent 10 g/L; room temperature).
(TIF)
