# Supplementary figures and images for: Removal of Vanadium(III) and Molybdenum(V) from Wastewater Using Posidonia oceanica (Tracheophyta) Biomass
Source: PLoS One. 2013 Oct 25;8(10):e76870. doi: 10.1371/journal.pone.0076870 (PMC3808364; doi:10.1371/journal.pone.0076870)

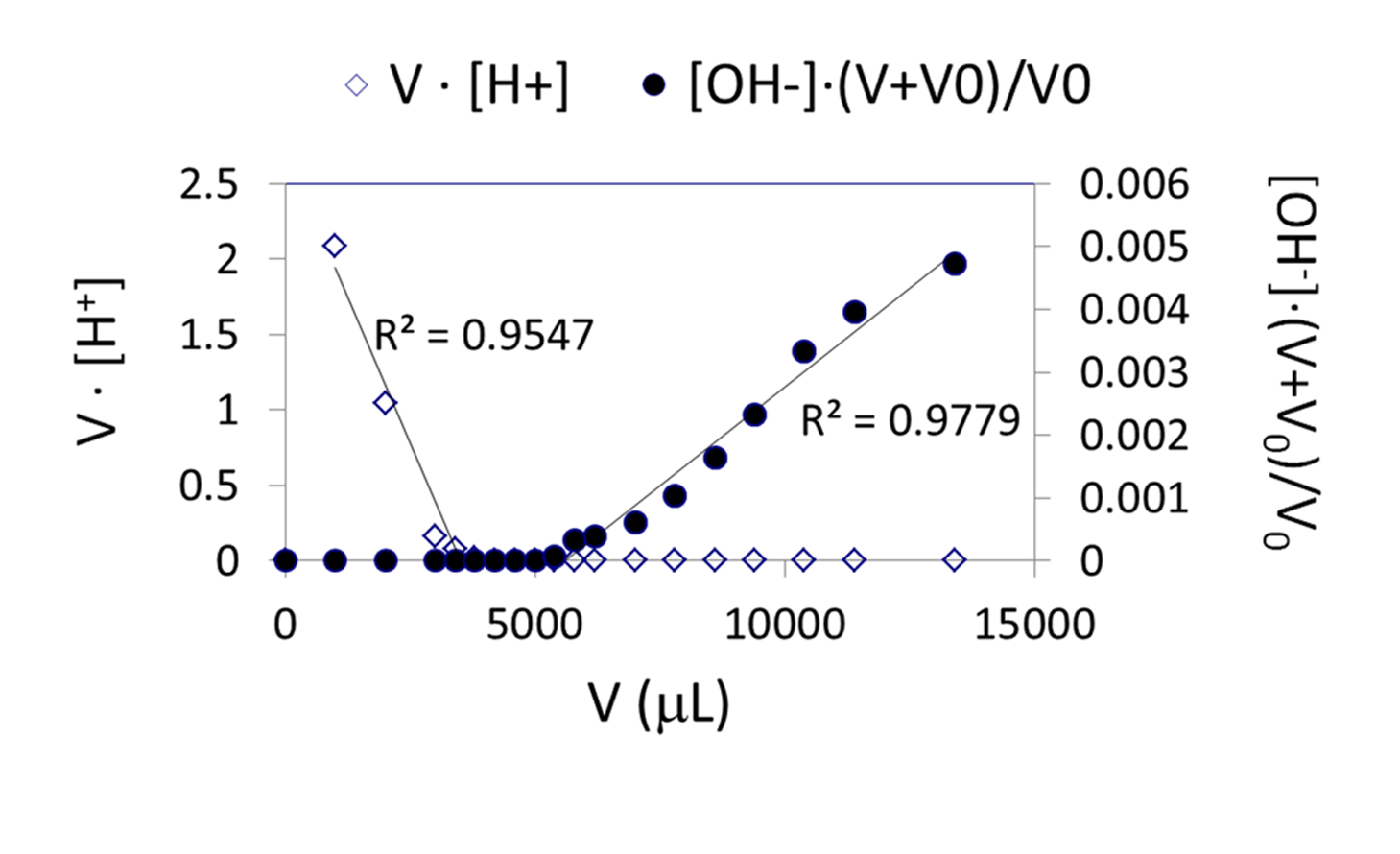

Supplement: Figure S1 — Gran elaboration of P. oceanica titration curve (biosorbent 10 g/L; room temperature). (TIF) [file pone.0076870.s001.tif]
